# The clean development mechanism in Eastern Europe: an in-depth exploration

**DOI:** 10.1007/s11356-022-20988-3

**Published:** 2022-05-31

**Authors:** José M. Cansino, Rocío Román-Collado, Sari Nassar

**Affiliations:** 1 Departamento de Análisis Económico Y Economía Política, Universidad de Sevilla, Avda. Ramón Y Cajal, 1. Postal Code, 41018 Seville, Spain; 2 Universidad Autónoma de Chile, Av. Pedro de Valdivia 425, 758-0150 Providencia, Santiago Chile

**Keywords:** Clean Development Mechanism (CDM), CDM projects, RES, UNFCCC, Eastern Europe, Kyoto protocol, Climate change

## Abstract

The Clean Development Mechanism (CDM), a partnership tool founded under the Kyoto Protocol, grants potential opportunities to help developing countries achieve sustainable development. The present research examines the CDM projects in Eastern Europe (Moldova, Serbia, Bosnia and Herzegovina, Montenegro, and Albania). Although there were far fewer projects in this region than, for instance, China or India, it has some specific features that make it worth studying. Major findings are that most CDM projects in Eastern Europe involved a changing combination of two or more sources of financing, and the distribution of projects in the region was uneven. Moreover, although there was a small number of projects overall, they were all cost-effective, long-term and large-scale. The findings of the research call for improvements to be made to the governance of the CDM, by strengthening the international and national regulation of projects and by aggregating the scales of decision-making and actions so that real multi-scalar transnational governance — from the global level down to the local level — is implemented in a coherent manner. It is also recommended to carry out ex-post project evaluations, following which readjustments could be made.

## Introduction

The Kyoto Protocol (KP) introduced a fundamentally new approach in the form of a cooperation tool aimed at reducing the costs associated with limiting greenhouse gas (GHG) emissions. Since climate change mitigation does not depend on where the emission reductions occur, a reasonable economic approach is to reduce them to the lowest possible level everywhere.

Accordingly, the KP provided three market mechanisms to maximize emission reductions: international emissions trading, joint implementation (JI), and what is known as the Clean Development Mechanism (CDM). In particular, the protocol stated that the CDM should help industrialized countries (those listed in Annex 1 of KP) to reduce the cost of meeting their emission reduction targets by implementing measures in other countries at lower costs than potential domestic costs (UNEP 2004; Huang and Barker 2012). The CDM acts as an international carbon trading mechanism linking Annex 1 (industrialized) countries to non-Annex 1 (developing) countries (Tang et al. 2022). The CDM offers countries and the private sector the opportunity to reduce GHG emissions anywhere in the world and count these reductions towards meeting their quantitative obligations (Jotzo and Michaelowa 2002; Cui et al. 2020; Benites-Lazaro et al. 2018). With the help of emission reduction projects, these mechanisms could stimulate international investment and ensure the flow of necessary resources for cleaner economic growth in all regions of the world.^1^

The significance of CDM projects has been raised by many researchers, including Hepburn (2007), Ruthner et al. (2011), Lückge and Peterson (2004) and Michaelowa and Dutschke (2002). Their research shows that there is still no unambiguous interpretation of the concept of the effectiveness of CDM projects and how to assess whether they fulfil their environmental prerequisites. Nor do we find a general methodological approach to CDM implementation in different areas and regions (Anger et al. 2007). Both in theory and in practice, CDM projects represent interaction within the framework of modern environmental cooperation.

The CDM projects aim to promote sustainable development in the host countries. However, the final decision on whether a particular project meets sustainable development criteria lies with the host country government. Adopted in 2015, the Sustainable Development Goals (SDGs) established a set of goals and targets to work towards in order to achieve sustainable development, focusing on the three dimensions of sustainable development: social, economic and environmental. The SDGs include a set of specific indicators for each target, which allow progress to be monitored in each case (The Danish Institute for Human Rights n.d.). The screening of CDM projects according to sustainable development criteria has the same aim. Specifically, from an economic and environmental perspective, SDG 7 and SDG13 are similar to the primary aims of CDM projects^2^ as they cover progress to more sustainable energy consumption and climate action. However, there are other goals linked to sustainable performance from a more general perspective that might be considered. Considering the results of CDM projects in terms of these objectives can help to assess their effectiveness. To do so, specific indicators linked to targets in SDGs which are related to the CDM can help to assess CDM projects.

From SDG 7 and 13, the relevant indicators are 7.2.1 renewable energy share in the total final energy consumption, 7.3.1 energy intensity measured in terms of primary energy and GDP, 7.b.1 installed renewable energy-generating capacity in developing countries (in watts per capita), 13.1.1 number of deaths, missing persons and directly affected persons attributed to disasters per 100,000 population and 13.1.2 number of countries that adopt and implement national disaster risk reduction strategies in line with the Sendai Framework for Disaster Risk Reduction 2015–2030. However, assessing the impact of the CDM projects analysed in terms of these indicators goes beyond the scope of this paper.

Notwithstanding a number of studies in the field of CDM projects and renewable energy sources (RES), there are few analyses of specific CDM projects in the countries under study here,^3^ despite the fact that this region is of particular interest as it has the highest energy intensity in Europe. Taking a closer look at the energy situation in this region confirms the scarcity of related scientific publications and research papers. Furthermore, this region is of special relevance considering that the geography of the region and its location in Europe sets the direction for the overall energy security of European countries (Lalic et al. 2011).

In view of the lack of research focused on Eastern Europe, the main aim of this work is to contribute to bridging this gap in the knowledge by examining various CDM projects in these countries, specifically, Moldova, Serbia, Bosnia and Herzegovina, Montenegro, and Albania. The region under investigation in our study was not chosen by chance. These countries are home to a considerable number of RES projects. Due to the critical proximity of these countries to the European Union (EU), they have a direct impact on the energy system of the European continent and its energy security.

The novelty of this paper lies in the methodological approach applied. The CDM projects are studied here not only from a financing perspective, but also taking into account the main economic and environmental features essential for the sustainable development of the host countries. To do so, the fundamentals of the projects and their goals are described in detail, the concept of “project efficiency” is analysed and the effectiveness of the projects is considered, including their economic and environmental efficiency. The analysis of the results of these projects in terms of economic and environmental efficiency is primarily based on statistical and financial data on the reduction of GHG emissions and quantitative indicators. Finally, this paper explores possible problems in the CDM decision-making process.

To the best of our knowledge, no previous papers have focused on this issue. The recent conflict between Russia and Ukraine has shone a light on European countries’ dependence on Russian gas. Given that many of the projects analysed in this research consist of replacing fossil fuel technologies, such as natural gas, with RES, the research is also of geostrategic interest.

The main findings show that most of the analysed CDM projects involved a changing combination of two or more sources of financing, and the distribution of projects in the region was uneven. Moreover, although there was a small number of projects overall, they were, without exception, all cost-effective, long-term and large-scale projects. The study highlights the contribution of these projects to sustainable development from an environmental and economic perspective.

The paper is structured as follows: After this introduction, the second section presents the literature review. The third section describes the method while the fourth section explains the data. The results are analysed and discussed in the fifth section. The conclusions are explained in the sixth section, along with some policy implications.

## Literature review

Studies of CDM projects tend to prioritize their temporal analysis and the interdependence between the result and the commencement date of the project (Liu et al. 2018). The measurement of various effects can be distorted over time, leading to inaccurate data and inconsistencies between the projected scenario and real emission reductions. Even when conducting a Cox regression analysis of the projects, it is worth paying attention to the additional calculations so as not to overlook some interdependent variables.

First of all, most CDM projects are effective in the short term, but ineffective in the long term, which casts doubt on this mechanism as a whole (Hepburn 2007; Cassimon et al. 2014). Within the sustainable development pathway, project indicator scores are very low, and the investment flow to these projects is targeted at a very narrow list of countries, which leads to geographic concentration of projects. A report published by the European Commission (Ruthner et al. 2011) conducts a thorough analysis of the current situation of CDM projects in Europe, addressing the policy from both the demand side and the supply side (Burtraw et al. 2007).

Fulfilment of KP commitments has posed challenges for countries to overcome, which may be solved by joining forces in the implementation of projects. The KP offered countries different mechanisms for sustainable development (Kiel Institute for World Economics 2004). The main distinguishing feature is the creation of national plans for EU members to build a more unified policy on the implementation of KP commitments. Countries with economies in transition receive investments from technologically developed countries (Alberola et al. 2008), primarily in the form of innovative technologies (Lowitzsch et al. 2020). However, despite the rapid development of the CDM system, the value of involving the private sector in project financing is rising (Michaelowa and Dutschke 2002). The CDM is seen as an opportunity to boost economic growth while reducing environmental emissions, that is, achieving the so-called net zero emissions economic growth in the framework of sustainable development. In other words, the CDM helps to break the link between economic development and environmental degradation (Koondhar et al. 2021).

The financing and construction of an energy facility (as well as its operation during the first few years) are assigned to a specially created project company. Nonetheless, it is worth noting that in recent years, the number of investments in the field of RES has been growing rapidly (Kirkman et al. 2013). Collective investment in such projects plays a prominent role in increasing the profitability of the project and improving its performance, but there is a lack of assessment and policy analysis of the environmental impact (Bossink 2017; Liu et al. 2019).

To achieve its climate goals, the energy market in Eastern Europe needs to transition from a system based on fossil fuels to a system based mainly on RES. First of all, the current balance of the state energy system must be examined, which will help to fully understand the general landscape and development paths of the country’s electrical production (Nikolakakis et al. 2019), as well as individual aspects, such as private sector optimization in energy consumption matters (Shankar et al. 2020).

The key to reducing emissions is improving efficiency in energy consumption. The advantages of integrating energy systems into a single energy system include achieving a more complete use of energy resources (Zhou et al. 2018; Acerbi et al. 2021; Kostakis and Tsagarakis 2022; Jahanger et al. 2022). CO_2_ emissions into the atmosphere can be reduced by changing both energy consumption and the electricity production system (Hawkes 2014).

One of the most important conditions for achieving global climate policy goals, preventing climate change as far as possible and adapting to and mitigating its consequences is the development of the innovative technology sector through international scientific and technical cooperation (Zhang and Yan 2015; Jiang et al. 2022). In this regard, climate agreements focus on the development and transfer of technologies (Das and Kasturi 2011), with a view to bridging the global technological gap. Indeed, such technologies play a central role in the ability to respond to the challenges associated with the negative effects of climate change (Gaast and Begg 2009). By involving developing countries in this partnership and better enabling their access to technology in the early stages of the technological cycle (Dixona et al. 2013), the conditions are being created for access to new environmentally-friendly technologies (Dixon et al. 2013). Such technologies should be introduced as soon as possible to help prevent climate change and adapt to the change that does occur (Gaast and Begg 2009).

Both market and non-market mechanisms play an important part in the transfer of carbon capture and storage (CCS) technology (Zakkour et al. 2014). The development of this technology facilitates the implementation of projects and the achievement of basic goals in primary scenarios for reducing GHG emissions (Dixon et al. 2013; Das and Kasturi 2011). Some organizations such as the World Bank and the Asian Development Bank have a significant role in promoting CCS in the framework of CDM (Lema and Lema 2013).

The objective of studies such as those by Manton et al. (2010), Chao and Feng (2018) and Burniaux et al. (2009) is to outline the most urgent global problems related to assessing the current climate situation and climate change projections in specific regions. This includes assessing the degree of anthropogenic impact on the climate (Hawkes, 2014); determining the main areas of climate research in developing countries, needed to prepare regional forecasts and economic and social development programmes; and presenting proposals on the climate doctrine concept (Manton et al. 2010). The factors driving climate change influence the flow of investments from Annex I countries to other countries where certain KP mechanisms might be implemented.

Papers that study the energy transition and the associated potential for Bosnia and Herzegovina can be divided into two groups. The first group examines the issue of RES in Bosnia and Herzegovina, a country which is certainly rich in such resources (Begić and Afgan, 2007; Karakosta et al. 2012). The second group specifically considers the strengths of the potential of Bosnian energy: hydropower and biofuels (Dogmus and Nielsen 2019).

In Albania, the main source of RES is hydroelectric power plants, although this entails energy problems during low tide and low water levels. Albania has a high potential for the development of RES (Xhitoni, 2013; Rickerson and Perroy 2005); however, it is worth noting that the strong points are biofuels, geothermal energy, and hydropower (Karaj et al. 2010; Frasheri 2013).

Over the past two decades, Serbia and Montenegro have made progress in the areas of RES and energy efficiency. Governments have developed various goals and policies to promote the use of RES in the region (Tešić et al. 2011). From a global perspective, however, their contribution remains negligible. For the Balkan region as a whole to reach the level of development of the global RES market, there is a need for increased investment flows and the implementation of related projects (Lalic et al. 2011).

Southeastern Europe accounts for a significant share of the continent’s RES potential. Albania, Bosnia and Herzegovina, Macedonia, Montenegro and Serbia have preferential tariffs in place to support their RES development goals (Komarov et al. 2012). National action plans have been approved in Montenegro and Serbia, as part of their membership of the Energy Community and in accordance with the requirements of compliance with EU Directive 2009/28/EC (Tešić et al. 2011). Wind energy accounts for a relatively large share of RES in Serbia and Montenegro (Mikicic et al. 2006) and is a fledgling source of RES in Serbia. In 2018, wind power provided 0.36% of the total electricity generated in Serbia, up from 0.15% in 2017 (Komarov et al. 2012). At the same time, Serbia has major potential for the energy use of biomass from agriculture and forestry (Cvetković et al. 2014).

The research on Moldova’s energy system pays special attention to heating (Gribincea 2013). The energy efficiency policy of Moldova is shaped by a combination of its energy problems and obligations due to its status of a member of the Energy Community. Moldova depends on energy imports, which provide 96% of its final consumption. The country receives financial support from several international donors for the development and implementation of energy efficiency regulation policies, including from the European Bank for Reconstruction and Development, the EU, and the United Nations Development Programme. Moldova occupies a leading position in the field of biogas and biofuels, in terms of the percentage they represent in the electricity generation sector. Non-economic barriers further drive up the cost of developing RES in the region (ŢÎŢEI 2002), while legal, administrative and institutional difficulties delay the implementation of related projects.

## CDM. Definitions and method

### CDM project implementation phases

**Fig. 1:**
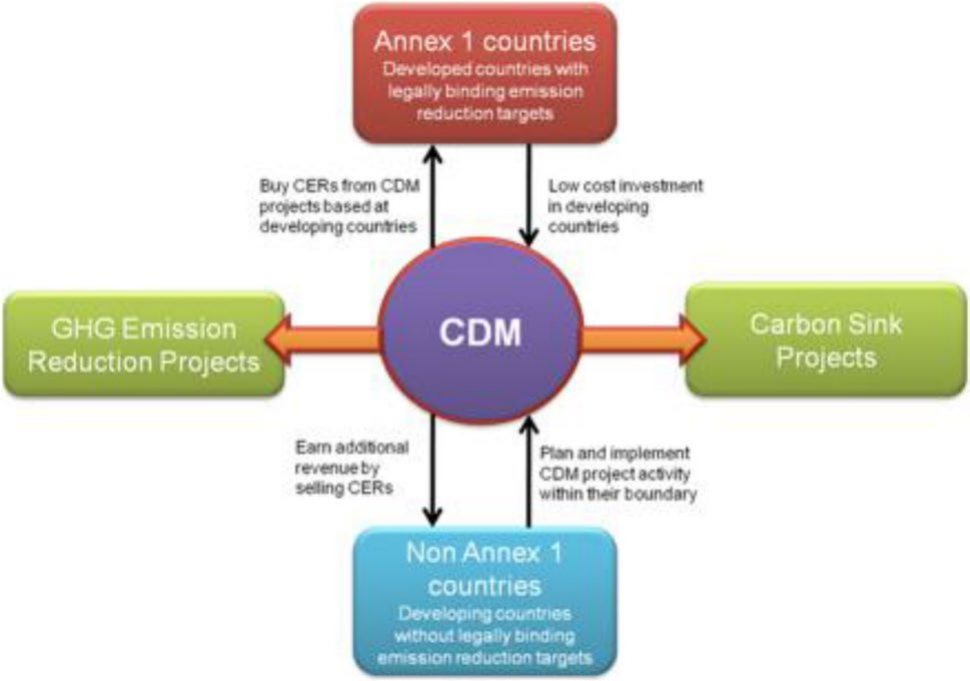
CDM architecture. Source: own elaboration

The CDM allows a party included in Annex 1 to implement a project to reduce GHG emissions or to remove GHG by absorbing carbon or promoting carbon sinks in the territory of a party not included in Annex 1 (Criqui and Kitous 2003). The resulting certified emission reductions (CERs) can then be used by the first party to offset its emissions in order to reach its emission reduction target (Convery 2009). CDM projects should be approved by all parties involved, lead to sustainable development in the host countries, and have a real, measurable and long-term effect on mitigating climate change. In addition, these emission reductions should be complementary to any reductions that could have been achieved without such a project. To participate in the CDM, countries must meet certain eligibility criteria (Burian 2006). All Parties must fulfil three basic requirements: voluntary participation in the CDM, designation of a national CDM body and ratification of the KP. Also, industrialized countries (usually Annex I) must meet several additional requirements: establishing certain quantitative obligations under Article 3 of the KP, having a national system for estimating GHGs, having a national registry, an annual inventory, and accounting systems for the acquisition or sale of emission reductions (UNFCCC 2012; Lee and Jang 2022; Dong et al. 2022; Huang et al. 2022). Figure 1 provides an overview of CDM architecture.

The CDM covers projects in the following seven sectors: (1) improving user-level energy efficiency; (2) improving energy efficiency in energy production; (3) RES; (4) fuel change; (5) agriculture (reduction of CH_4_ and N_2_O emissions); (6) industrial processes (CO_2_ in cement production, etc., HFCs (hydrofluorocarbons), PFCs (fluorocarbons), SF_6_ (sulphur hexafluoride), NF_3_ (nitrogen trifluoride)); and (7) absorption projects (afforestation and reforestation only). To ensure the competitiveness of small-scale projects in comparison with large-scale projects, the Marrakesh Accords provide for a simplified procedure with less stringent acceptance criteria. The CDM also covers the use of RES up to 15 MW, or energy efficiency with a lower consumption (either on the production side or on the consumption side) up to 15 GWh/year, and other project activities that both help reduce emissions and directly emit less than 15 thousand tonnes of CO_2_ equivalent (CO_2eq_) per year.

With partial or complete state funding of CDM projects, funds allocated for official development assistance should not be used. Besides, CERs received through CDM projects are subject to a 2% fee, known as a “share of the proceeds”, which is paid to the newly created adaptation fund to help the most vulnerable developing countries adapt to the negative effects of climate change. Another CER fee is used to cover the administrative costs of the CDM. To facilitate the equitable distribution of projects among developing countries, CDM projects that are implemented in the least developed countries are exempt from the fees payable to the adaptation fund and to the administrative costs fund.

The implementation of the CDM is overseen by the Executive Board, which is led by the parties. The Executive Council consists of 10 members, including one representative from each of the five official UN regions (Africa, Asia, Latin America and the Caribbean, Central and Eastern Europe and the OECD), one delegate from small island developing countries, and two representatives from each Annex I country and each non-Annex I country. The Executive Council held its first meeting during the negotiations in Marrakech (November 2001), which marked the launch of the CDM.

**Fig. 2:**
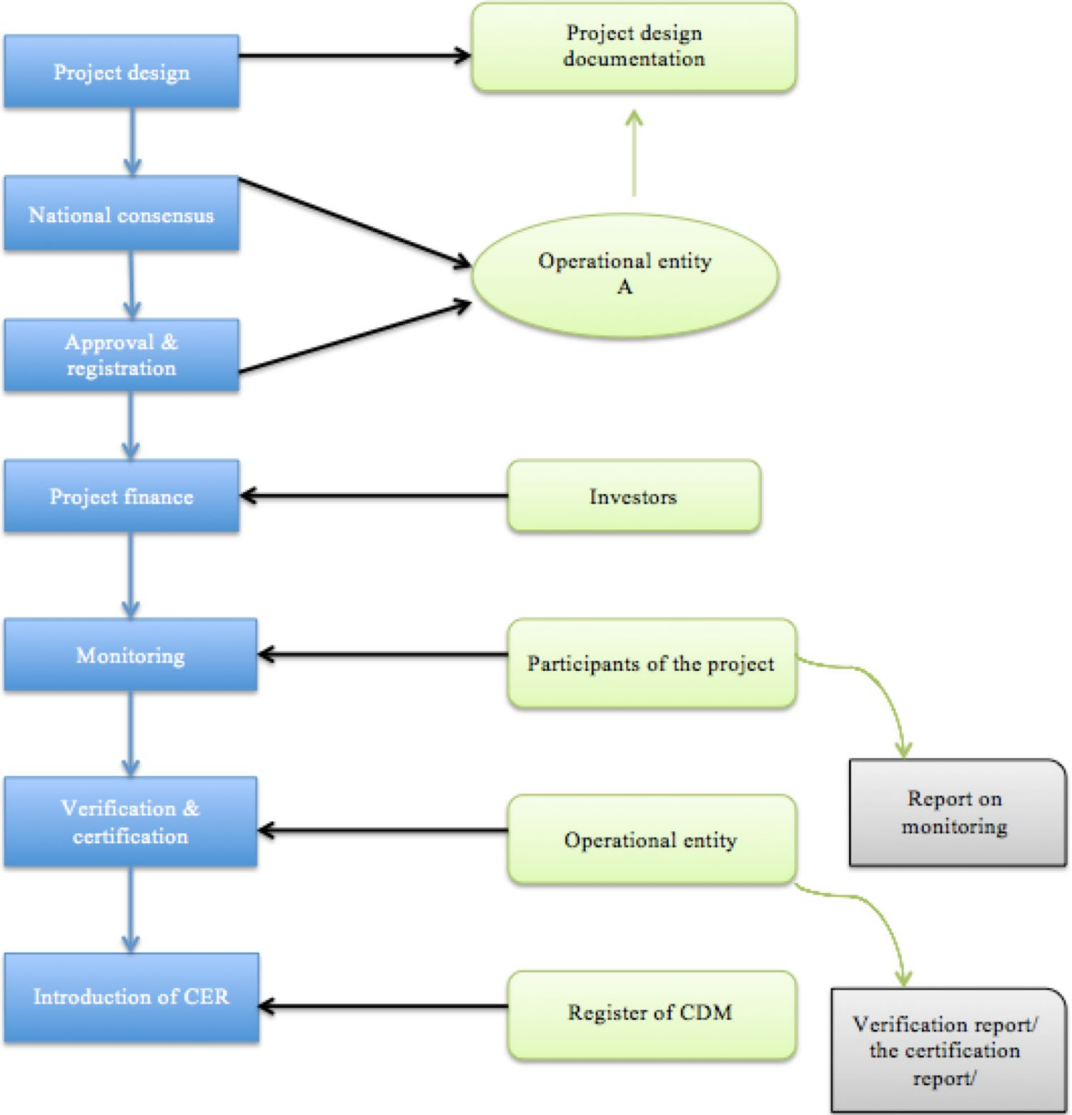
Project cycle under the CDM. Source: own elaboration

Figure 2 details the CDM project cycle. It consists of seven stages: formulation and development of the project, obtaining national consensus, approval and registration of the project, financing of the project, monitoring, verification and certification and issuance of CERs. The first four stages of this cycle are carried out before the project gets underway, while the latter are associated with the operational period of the cycle. The blue boxes indicate the actions of the cycle, while the green ones are participants and reports during the cycle. More detailed information is provided in Annex A of this paper.

### Method

An extensive literature review on the subject of CDM projects was carried out using the main databases of scientific documents, primarily official United Nations sources, in particular UNFCCC. An additional source was Web of Science for scientific publications on the topic, as well as topics related to the issue under analysis in this research. To the extent possible, aspects relating to the countries under analysis are explicitly highlighted whenever available data or information exists.

We then identify and provide details on CDM projects conducted during the analysed period, which varies from country to country but may be defined as covering 30 years (2006–2036). It is worth noting that there is a limited amount of peer-reviewed literature about this topic.

This research was conducted using a combination of approaches and scientific methods. The abstract-logical method was used to reveal the theoretical aspects of assessing the financial condition and financial stability of projects, in order to determine the main characteristics of the processes and phenomena in this area. The system-structural method was used to analyse the financial condition and identify structural changes.

**Fig. 3:**
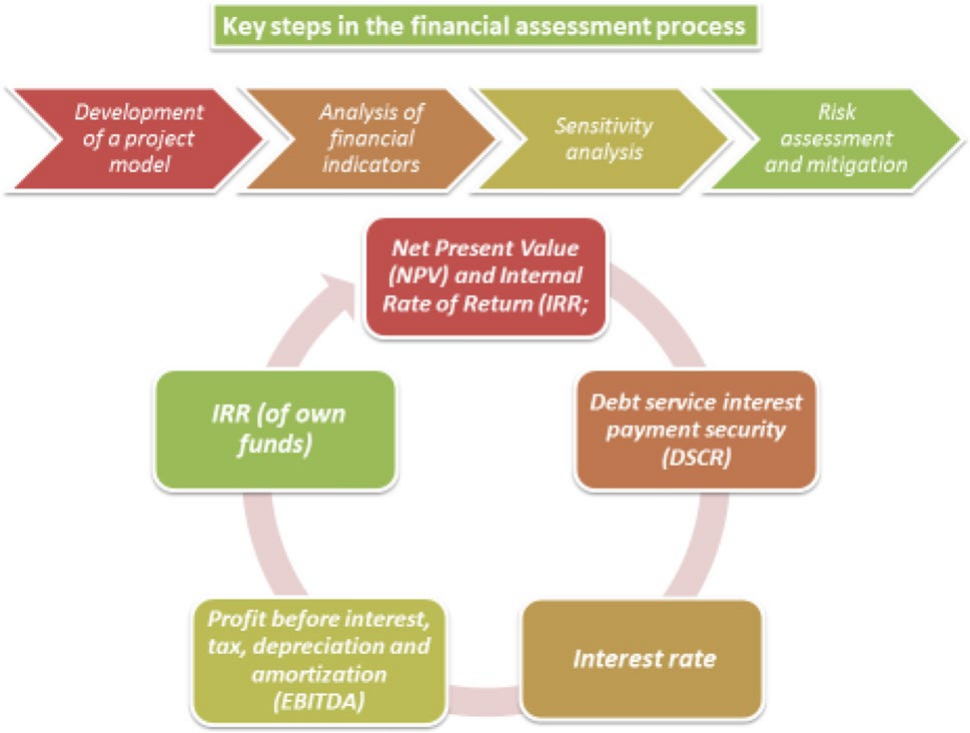
Key steps in the financial assessment process. Note: EBITDA, earnings before interest, taxes, depreciation, and amortization; IRR (equity), internal rate of return on equity investment; DSCR, debt service cover ratio. Source: own elaboration

The financial appraisal process is a standard approach for assessing the economic viability and environmental efficiency of a project (see Fig. 3). The financial evaluation of the project is part of the “ in-depth audit” carried out by the investor or as part of the general research process for the proposed investment. The in-depth audit process should also include an evaluation of the ability of the management team to complete the project, an investigation into the technology to be used and ongoing monitoring of the project after funding. Here, however, we focus on the financial evaluation of the process, pre-financing.

Although detailed financial data, such as monthly cash flow reports, provide the necessary information to evaluate project performance, some different indicators can also be used to summarize the situation. The relative importance of various indicators differs between debt and equity providers, although the basic principles are the same. The indicators commonly used are the following: (1) net present value (NPV) and internal rate of return (IRR); (2) IRR (equity); (3) earnings before interest, taxes, depreciation and amortization (EBITDA); (4) interest rate and (5) debt service cover ratio (DSCR). However, in our research, certain indicators were not used due to a lack of data and/or the fact they were not relevant to the studied projects. Thus, NPV and IRR and IRR (equity) were considered in the analysis, while EBITDA, interest rate and DSCR were not applicable to the projects under study here.

It is important to emphasize the indispensable role of NPV and IRR when it comes to assessing CDM projects in general, and our analysed projects in particular. The aforementioned indicators are needed to ascertain the cost-effectiveness of investment projects. Moreover, they tell us the cost–benefit the project provides in comparison with other financing alternatives for a similar period. Detail on each country is presented in Annex B.

## Data

The data used were sourced from the official UNFCCC database, UNdata, the Open SDG Data Hub, MBS, the UN Comtrade database, the National accounts – Analysis of Main Aggregates (AMA) and the UN Digital Library. After a systematic search of the whole set of aforementioned sources, the projects for analysis were hand-selected.

As mentioned above, the main data source is the official UNFCCC database. The CDM page of the UNFCCC website has a “Project Search” tab. On this page, in the public domain, the search tool makes it possible to find specific CDM projects as well as general project types. The UNFCCC database contains all the projects that are at various stages of the registration process, as well as rejected projects.

The search tool has a user-friendly interface, which offers various ways to search for a project: by name, for instance, or using the project classification. Furthermore, all projects are grouped by their size (large or small) and can be at any stage of adoption, which may be indicated. A search can also be conducted by reference number, if known. Another significant advantage of the system is the advanced search function, which enables the user to select or reject projects according to the methodology applied, as well as the host country. It also makes it possible to indicate the date of registration, and the amount of emission reductions. It is worth noting that the database includes a wide range of methodologies used for calculating and submitting projects, where the user can find a large amount of information regarding particular projects, as well as methodological recommendations in general. The register of CDM projects also provides information on the evaluation and status of existing as well as completed projects. Lastly, the UNFCCC database provides information on “Investor Interaction” to study current projects and trends in capital flow through CDM partnerships.

Another essential source is UNdata. This is a search system that provides access to data from UN system databases. UNdata began operations in February 2008 and is the outcome of a partnership among the UN Statistics Division, the Swedish Statistical Office and the Swedish International Development Cooperation Agency. UNdata enables the user to explore and download data from many statistical resources, covering such subjects as energy, the environment, employment, food and agriculture, health, human development, industry, information and communication technologies, national accounts, population, refugees, commerce and tourism. On the official UNdata website, the user can find an enormous amount of statistical information, reports, paperwork, statements and infographics from all UN entities, providing genuine and unique data. The data are divided into datasets, sources and topics, which makes it easier to find the desired information. In addition, there is an “Update Calendar” section, which simplifies the separation of data into dataset, source, organization, last update and next update; this feature proves extremely useful for tracking down the needed information. Also helpful is the glossary, which provides the user with official definitions of key terms.

Furthermore, it is worth highlighting the other databases used in our study: the Open SDG Data Hub, MBS (monthly bulletin of statistics online), the UN Comtrade database, the National accounts – Analysis of Main Aggregates (AMA) and the UN Digital Library.

## Results and discussion

The CDM projects are studied here from a financing perspective, but we also take into account the main economic and environmental features. The results of this analysis allow us to discuss both the local and global relevance of these projects.

### Types of financing of CDM analysed

From a financing perspective, there are usually three types that can be used to develop CDM projects: grants, loans (debt), and equity. However, most CDM projects will involve a changing combination of two or more sources of financing, due to the large number of necessary investments. While there are some typical models of project financing that were considered in this research, it should be noted that not all of them were applied to the specific projects here, due to certain characteristics of our chosen region. Thus, project financing (in a particular sense of this term), also known as limited recourse financing, corporate financing and leasing financing, was utilized in the projects in question.

Although some less common financing types were not used (e.g., interim financing, microloans, collateral financing), one of the most recent and relevant types was implemented — namely, Energy Service Company/Renewable Energy Service Company (ESCO/RESCO) (see Table 1) — which has proved fundamental to the projects analysed here. Since they are focused on such sectoral scopes as energy industries, RES, energy distribution and energy demand, these projects would not be feasible without ESCO/RESCO. Table 1 below details three key types of financing.

**Table 1:** Main types of financing structures

Types	Features	Projects
(i)Project financing by an Independent Energy Producer	Project financing is often used for projects by independent energy producers To complete the project, project sponsors must establish a subsidiary specialized enterprise (SE). The project sponsors should provide the initial US$ 2 million for the planning phase as an equity investment. The Joint Venture (JV) will sign a long-term (for example, 15 years) Energy Purchase Agreement (EPA)	Moldova Biomass Heating in Rural Communities 2; Moldova Energy conservation and GHG emissions reduction; HPP Ulog (Bosnia); HPP Ashta (Albania); Devoll Hydropower (DHP) (Albania); Hydropower station Murdhari 1&2 (Hydroelectric Power Station Murdhari (Albania); Moldova Community Forestry Development Project
(ii)Corporate financing for an energy efficiency project	Implementation of the project saves the company money (by reducing the cost of energy, say by US$1 million/year). If the investment is well planned and the company is large enough, the company may be able to finance such a project directly from its funds. Alternatively, a company may borrow part of the capital from a bank (or a syndicate of banks), with its main assets as collateral for a loan, provided that the company is sufficiently creditworthy	Moldovagaz, Amitea Small Hydro Project (Bosnia); LFG Recovery and Electricity Production at the Bubanj Landfill Site (Nis, Serbia); and Alibunar Biogas plant construction project (Serbia)
(iii)ESCO/RESCO CDM projects	ESCOs are typically used to support demand-side energy efficiency projects where the result of the investment is energy conservation for the consumer. Since the consumer may not have the desire (or financial ability) to make energy-saving investments, the ESCO may propose to complete the project, receiving income from the consumer proportional to energy conservation, as established in the framework of the Energy Regime Contract. RESCOs are commonly used to power rural areas in developing countries using RES	Hydropower Plant Otilovici and Mozura Wind Farm (Montenegro); Wind Farm Cibuk 1 (Serbia); Wind Farm Plandiste 1(Serbia); Wind Farm Kosava I + II (Serbia); Wind Farm Kladovo 1 (Serbia)

Source: own elaboration.

### The selected CDM initiatives by country

Globally, 8026 projects that comply with all of the CDM criteria and rules have been “registered” with the UNFCCC, as of 30 April 2020. Of these projects, 2291 effectively transformed the reported GHG reductions into carbon credits. A total of 1656 million tonnes of CO_2eq_ were reduced between 2004 and April 2016 and transformed into carbon credits (Mendez-Sayago and Perugache-Rodriguez 2012; Mansanet-Bataller et al. 2011; Vasa 2012; Zhang et al. 2018). It is important to highlight that all the registered projects from Eastern Europe are analysed here.

Table 2 lists the identified CDM projects in the countries under analysis. The total number of projects in these countries is quite small. It is worth noting some features of individual countries and their projects. The projects in Moldova have large financial flows and ambitious annual emission reductions, which certainly make these projects cost-effective. Moldova’s projects are mainly related to energy derived from biofuels, or the modernization of the country’s gas system.

**Table 2:** Main data of the analysed CDM projects

Country	Project name	Emissions avoided	Entity List	Total investment	Total investment / Annual emissions reduction ($ per tCO_2eq)_
Moldova	**Moldova Biomass Heating in Rural Communities 2 (01 Jan 2008–31 Dec 2017)**	-Annual average over the crediting period of estimated reductions: 5781 tCO_2eq_ -Total (real) emissions reduction: 57,810 tCO_2eq_	**-*Annex C***	US$ 7,654,302.00	7,654,302.00 / 5781 = 1324.04
	**Moldova Energy conservation and greenhouse gas emissions reduction (29 Jan 2006–29 Jan 2016)**	-Amount of reductions: 11,567 tCO_2eq_ -Total (real) emissions reduction: 61,156 tCO_2eq_	**-*Annex D***	US$ 3,993,000.00	3,993,000.00 / 11,567 = 345.21
	**Reducing gas leakages within Moldovagaz distribution network, Republic of Moldova. (01 Jan 2013–31 Dec 2022)**	-Annual average over the crediting period of estimated reductions: 748,903 tCO_2eq_ -Total (real) estimated reductions: 7,489,030 tCO_2eq_	-Republic of Moldova (Host)—Moldova-Russian Joint Stock Company Moldovagaz (energy company) -Denmark — Danish Carbon Holding ApS (Consulting engineering activities within construction) -Denmark — Nordjysk Elhandel A/S (energy trading and management company)	US$ 1,482,806.00	1,482,806.00 / 748,903 = 1.97
	**Moldova Community Forestry Development Project (01 Nov 2006–31 Oct 2036)**	-Total (real) estimated reductions: 1,171,708 tCO_2eq_	-Republic of Moldova, involved indirectly, Agency Moldsilva; International Bank for Reconstruction and Development as trustee of the BioCarbon Fund -Spain, involved directly, Kingdom of Spain — Ministry for the Ecological Transition & Ministry of Economy and Business; Zeroemissions Carbon Trust, S.A.; International Bank for Reconstruction and Development as trustee of the BioCarbon Fund -Ireland involved directly, Government of Ireland, Department of Communications, Climate Action and the Environment	US$ 27,000,000.00	27,000,000.00 / 39,056 = 691.32
Montenegro	**Hydropower Plant Otilovici (01 Jul 2014–30 Jun 2021)**	-Annual average of the estimated emission reductions over the crediting period: 13,200 tCO_2eq_ -Total (real) estimated reductions: 92,400 tCO_2eq_	-Montenegro, involved indirectly, Elektroprivreda Crne Gore AD Nikšić (integrated electricity company, the majority of whose shares are state-owned) -Italy, involved indirectly, A2A S.p.a. (utility company)	US$ 5,745,600.00 *(€4,200,000 – exchange-rate €/US$: 1.37, year 2014)*	5,745,600.00 / 13,200 = 435.27
	**Mozura Wind Farm (01 Jan 2014–31 Dec 2020)**	-Annual average of the estimated emission reductions over the crediting period: 79,632 tCO_2eq_ -Total estimated reductions: 557,421 tCO_2eq_	Montenegro (host) — Mozura Wind Park, D. O. O. (Private entity) (Production of electricity) United Kingdom — CO_2_ Global Solutions International S.A. (Private entity) (business consulting and others)	US$ 87,497,363.60	87,497,363.60 / 76,632 = 1141.79
Bosnia and Herzegovina	**Amitea Small Hydro Project (01 Jan 2014–31 Dec 2020)**	-Annual average of the estimated emission reductions over the crediting period: 37,158 tCO_2eq_ -Total estimated reductions: 260,106 tCO_2eq_	Bosnia and Herzegovina (host) — Amitea d.o.o. Mostar (private entity) (Electric Utilities Industry) France — Solvay Energy Services SAS (private entity) (advanced materials and specialty chemicals)	US$ 37,831,026.40 *(€27,499,474—exchange-rate €/US$: 1.38, year 2014)*	37,831,026.40 / 37,158 = 1018.11
	**Hydro Power Plant Ulog (01 Oct 2017–30 Sep 2027)**	-Annual average of the estimated emission reductions over the crediting period: 87,846 tCO_2eq_ -Total estimated reductions: 878,460 tCO_2eq_	Bosnia and Herzegovina (host) — EFT HE Ulog d.o.o. (Energy trading and investment firm)	US$ 49,108,908.70 *(€41,702,538—exchange-rate €/US$: 1.18, year 2017)*	49,108,908.70 / 87,846 = 559.03
Albania	**HPP Ashta (01 Mar 2013–29 Feb 2020)**	-Annual average of the estimated emission reductions over the crediting period: 78,989 tCO_2eq_ -Total estimated reductions: 465,311 tCO_2eq_	Albania — Energji Ashta Shpk (electricity company); VERBUND Hydro Power AG (electricity, hydropower); EVN AG (international energy company) Austria — VERBUND AG	US$ 245,361,535.00 *(€188,334,000 exchange-rate €/US$: 1.30, year 2013)*	245,361,535.00 / 78,989 = 3106.27
	**Devoll Hydropower (DHP) (01 Jul 2020–30 Jun 2030)**	-Annual average of the estimated emission reductions over the crediting period: 339,052 tCO_2eq_ -Total estimated reductions: 3,390,520 tCO_2eq_	Devoll Hydropower Sh.A. (DHP); EVN AG (international energy company); Statkraft AS (hydropower company)	US$ 854,655.62 (*€791,347.80—exchange-rate €/US$: 1.08, year 2020*)	854,655.62 / 339,052 = 2.52
	**Hydropower station Murdhari 1&2 (Hydroelectric Power Station Murdhari in Albania), (13 May 2015–12 May 2025)**	-Annual average of the estimated emission reductions over the crediting period: 5,807 tCO_2eq_ -Total estimated reductions: 58,078 tCO_2eq_	Hydroenergy Sh.p.k., Triana (private entity) (hydropower for electricity)	US$ 11,819,083.00 *(€10,490,000 exchange-rate €/US$: 1.13, year 2015)*	11,819,083.00 / 5807 = 2035.32
Serbia	**Wind Farm Cibuk 1 (01 Jan 2013–31 Dec 2019)**	-Annual average of the estimated emission reductions over the crediting period: 499,967 tCO_2eq_ -Total estimated reductions: 3,499,770 tCO_2eq_	-Government of Serbia (host) -Vetroelektrane Balkana d.o.o. (Private) (Producer of wind energy.) -Government of Liechtenstein -Energy Changes Projektentwicklung GmbH (private)(consulting and engineering company) Plus Ultra Asset Management GmbH (private) (Financial Planners & Investment Advisers Industry)	US$ 396,090,000.00 *(€300,000,000 exchange-rate €/US$: 1.32, year 2013)*	396,090,000.00 / 499,967 = 792.23
	**Wind Farm Plandiste 1 (01 Jan 2013–31 Dec 2019)**	-Annual average of the estimated emission reductions over the crediting period: 332,241 tCO_2eq_ -Total estimated reductions: 2,325,685 tCO_2eq_	-Government of Serbia (host) -Wind Park Plandiste doo (private) -Government of Liechtenstein—Energy Changes Projektentwicklung GmbH (private)(consulting and engineering company), Plus Ultra Asset Management GmbH (private) (financial Planners & Investment Advisers Industry)	-	-
	**Wind Farm Kosava I + II (30 Sep 2013–20 Sep 2020)**	-Annual average of the estimated emission reductions over the crediting period: 459,622 tCO_2eq_ -Total estimated reductions: 3,217,357 tCO_2eq_	-Government of Serbia (host) -MK-Fintel Wind AD (private) (renewable energy company) -Government of Liechtenstein -Energy Changes Projektentwicklung GmbH (private)(consulting and engineering company), Plus Ultra Asset Management GmbH (private) (Financial Planners & Investment Advisers Industry)	-	-
	**Wind Farm Kladovo 1 (01 Oct 2013–30 Sep 2023)**	-Annual average of the estimated emission reductions over the crediting period: 130,538 tCO_2eq_ -Total estimated reductions: 1,305,379 tCO_2eq_	-Serbia (host) — Forestpeak-I d.o.o. (Private) -Liechtenstein — Energy Changes Projektentwicklung GmbH (private) (consulting and engineering company),	-	-
	**LFG Recovery and Electricity Production at the Bubanj Landfill Site, Nis, Serbia (01 Jan 2014–31 Dec 2023)**	-Annual average of the estimated emission reductions over the crediting period: 13,415 tCO_2eq_ -Total estimated reductions: 134,155 tCO_2eq_	-Republic of Serbia (Host Country) — AMEST doo -Italy — AMEST S.r.l. (private entity)	US$ 1,306,630.00 (€*950,000 exchange-rate €/US$: 1.38, year 2014)*	1,306,630.00 / 13,415 = 97.4
	**Alibunar Biogas plant construction project (01 Aug 2013–31 Jul 2023)**	-Annual average of the estimated emission reductions over the crediting period: 13,415 tCO_2eq_ -Total estimated reductions: 134,155 tCO_2eq_	-Republic of Serbia (host) Biogas Energy d.o.o. (Private Entity) (Bioenergy company) -United Kingdom of Great Britain and Northern Ireland—Camco Carbon International Limited (Private Entity) (financing and building clean energy and electrification projects)	US$ 19,124,765.90 *(€14,458,884 exchange-rate €/US$: 1.32, year 2013)*	19,124,765.90 /13,415 = 1425.63

Considering Montenegro’s projects, there are only two that were highly effective in terms of the ratio of investments to the annual emission reduction, and they required major investments. The first project was carried out in partnership with an Italian company; the second was one of the most expensive projects, in partnership with the UK. Both projects were wind parks. Bosnia and Herzegovina also had two projects, both of which were hydroelectric. As with the Montenegrin projects, the ratio of investments to the annual reduction of CO_2_ emissions was fairly high, which made these projects very environmentally cost-effective.

The three Albanian projects were also carried in the field of hydropower. It should be noted that the HPP Ashta project had the greatest environmental cost-effectiveness of all the projects, with a ratio of 3106.27 (ratio of investments/annual reduction of CO_2_). Most of the Albanian projects were handled in partnership with Austrian energy companies. All projects had fairly high investment inflows, as well as reasonably high annual emission reductions.

It is important to note the ambitious biogas projects, for example, in Moldova and Albania. We should also point out the large, arduous projects in the field of hydropower. Since the region has great potential in the field of hydropower, a substantial number of projects were developed in Bosnia and Herzegovina and Albania. It should be noted that both projects in Bosnia and Herzegovina showed very high cost-effectiveness; these projects naturally require more investments than wind power projects for example. Nevertheless, the average annual reduction in CO_2_ emissions is also quite high, which made them more environmentally friendly. This allowed these countries to more actively pursue energy integration policies.

Serbia had the largest number of projects; however, half of them did not provide detailed investment information, which made it challenging to evaluate their profitability. Most were projects in the field of wind energy and only one was in the field of biogas. Moreover, the biogas project was one of the most environmentally effective projects in terms of the environment in general. The main partners with whom these projects were implemented were companies from the UK, Italy and Liechtenstein.

**Fig. 4:**
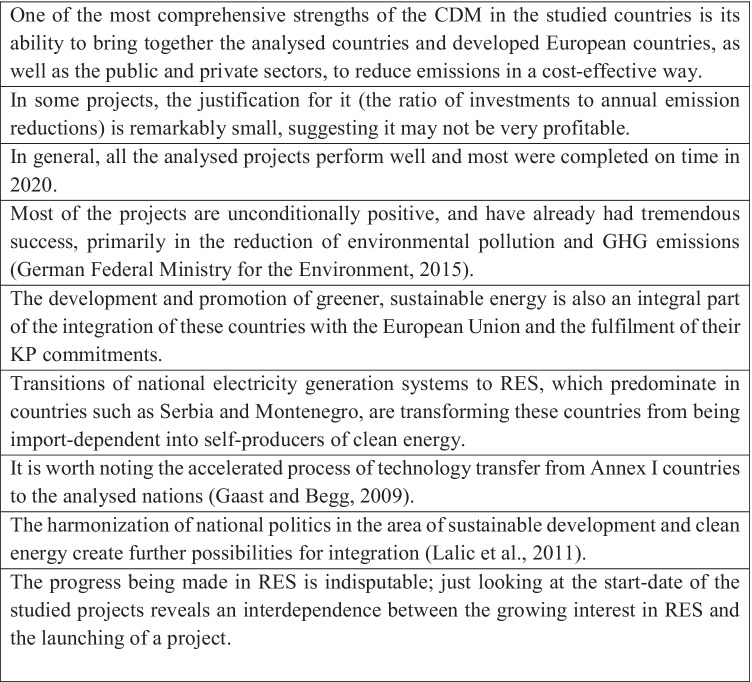
Main features of identified CDM projects. Source: own elaboration

To sum up, the analysis of the projects in Eastern European states is summarized in Fig. 4 below.

All the analysed countries expressed interest in the negotiation process within the UNFCCC and advocate for the adoption of a legally-binding accord to prevent the Earth’s average temperature from rising by more than 2 °C. These countries have also confirmed their targets for emissions reductions by 2030 (Serbia to reduce emissions by 10% by 2030; Montenegro 30% by 2030; Bosnia and Herzegovina 2% by 2030; Albania up to 12% by 2030; Moldova up to 70% by 2030) (Djurovic et al. 2019; Udovicki et al. 2018; Report of Government of the Republic of Moldova, March 2017; Report of the Bosnian Government, April 2019). In the energy balance of these countries, a significant part of primary consumption is already covered by RES, contradicting the environmental Kuznets curve (EKC) hypothesis. The mechanisms of the KP, including the CDM, as well as their growing integration with the EU, has played a decisive role in this process. That said, the analysis of the behaviour of the analysed countries during the implementation of CDM projects in their territory revealed that, within this region, government support varies from country to country. This led to an uneven distribution of projects in the region, and a small number of projects overall, despite the region’s high potential in the field of RES.

The CDM projects analysed undoubtedly make an essential contribution to achieving sustainable development, meaning the projects of the analysed region are aligned with the SDGs (Ugochukwu 2020; Usman and Balsalobre-Lorente 2022). Since they are almost all projects in the field of RES, they contribute to the achievement of several different SDGs,^4^ such as SDG7 (affordable and clean energy) and SDG13 (climate action) (Djurovic et al. 2016). Sustainable and renewable energies are key to sustainable development, not only from an environmental perspective but also from an economic and social one. Firstly, because these energy sources allow countries to reduce their dependence on fossil fuels, lowering the risks due to the volatility of prices and quantities caused by possible energy shocks. Secondly, they help to improve the balance of payments of the economy, since they reduce the need for imports of fossil fuels. And thirdly, unlike fossil fuels, they are low-pollution or pollution-free energy sources, helping to reduce the stress on the environment generated by businesses and the residential sector/household consumption of energy. For all these reason, the boost to RES thanks to CDM projects in the developing countries analysed facilitates progress towards sustainable development.

Additionally, if the implementation of CDM projects was accompanied by complementary information and data about the social and economic impacts, it would help to identify the progress made towards a global concept of sustainable development. The analysis carried out shows that the studied projects have undeniably contributed to the countries’ performance in terms of SDG3 (good health and well-being), SDG8 (decent work and economic growth), SDG9 (industry innovation and infrastructure), and SDG17 (partnerships for the goals). After all, one of the aims of the CDM is to provide a cheaper solution for developing countries to achieve their targets.

Following the analysis of the studied projects, the initial purpose of the CDM to function as an effective climate finance tool can clearly be seen in the individual projects (Olsen 2007). By analysing the financial data from the projects (where the information was available), the main inference drawn is that all projects, without exception, were cost-effective, long-term and large-scale (in relation to the size of the country’s economy and its energy system). Moreover, they were collaborative, which, in addition to the inflow of investments from Annex 1 countries, helped to accelerate the process of technology transfer. All projects were financially viable and suitable for investment and financing. Each project had a relatively short payback period (within the industry).

Most projects conducted a sensitivity analysis considering various possible scenarios. Within the framework of its sensitivity analysis, each project justified its cost-effectiveness given its inherent uncertainty and risks. It has been shown that the projects under consideration have a relatively low sensitivity, which makes it easier to interpret the economic and environmental indicator of annual emission reductions.

The last finding relates to an assessment of the environmental role of the projects. Each project was under the supervision of a national environmental review, which took into account legislative requirements in the field of environmental impact assessment. Each project (with the exception of three projects in Serbia) conducted an evaluation of the hypothetical damage and benefits, within the framework of the integrated environmental and economic effectiveness analysis.

## Conclusions

This research allows us to conclude that, in a short period of time, the CDM acted as a catalyst for a large number of project activities in Eastern Europe. All the selected projects supported decarbonization processes. Without this mechanism, it is difficult to imagine such a result would have been achieved. In spite of the overall progress driven by the CDM, the outcomes have not always been entirely clear or acknowledged by all actors. The benefits in terms of sustainable development (SDGs), technology transfer, additionality of funding and global emission reductions have been called into question, as has the fair distribution of the benefits (the CDM market has been dominated a few non-Annex 1 parties).

The contribution to the sustainable development of host countries was one of the two main objectives of the CDM. It is therefore important to assess the impact of CDM projects in terms of sustainable development. This evaluation constitutes a prerequisite for the acceptance of projects by the Executive Council, but it must be said that the requirements in this area are extremely limited. In fact, to meet the requirements of the Executive Council, it is enough for the host country to certify that the project complies with its sustainable development policy. One might therefore argue that there is no reason to further question the principles and methods which should guide the ex-ante evaluation of CDM projects from the point of view of sustainable development. This would be to ignore the fact that, in the absence of an explicit national policy of sustainable development, host countries may be anxious to verify that the projects submitted to them nevertheless fall within this scope. Moreover, Annex 1 countries may wish to impose additional admissibility criteria on the projects presented to their National Authority, including in matters of local development.

To ensure the success of CDM in the future, it is important to make prompt modifications to governance, market functioning and project scope. Adjustments performed in the short term are best done in the context of a strategic outlook with plainly defined goals. Such changes must be meaningful enough to produce continuity and to rebuild reliance on the UNFCCC’s ability to implement the mechanism. This will be especially challenging in light of developments in the negotiations over the medium term.

It seems essential to improve the governance of the CDM by strengthening the international and national regulations of projects and by aggregating the scales of decision-making and actions, so that real multi-scalar transnational governance — from the global level (CDM Executive Council) down to the local level (places where projects are carried out) — is implemented in a coherent manner. It also crucial to improve the effectiveness of projects, by carrying out ex-post evaluations, following which readjustments could be made.

Despite the shock caused by the SARS-CoV-2 pandemic, nations are still striving to reduce emissions, while overcoming the crisis and developing their economies in a sustainable way. An effective battle against climate change requires much more drastic emission reductions by the world’s top emitters. By drawing on these lessons from the past and involving civil society at the heart of CDM governance, it will surely be possible to produce a robust and adaptive institutional framework.

## Data Availability

Upon reasonable request.

## Annex 1. Project cycle under the CDM. Details

### Project design

The first step in the CDM project cycle is the selection and development of potential projects. The CDM project should be realistic, measurable, and complementary. To establish the additional nature of the project, the emissions associated with it should be compared with the emissions of the most reasonably probable development of the action, which are called baseline conditions. The project participants set these initial conditions using the approved methodology for each specific project. These emission baseline methodologies are being developed following the three approaches from the Marrakesh Accords:

- Existing actual or historical emissions;
- Emissions resulting from the use of technology, which is an economically attractive line of business, taking into account barriers to investment; or
- Average emission standards as a result of similar project activities, which were carried out in the previous five years under similar conditions and which, according to all indicators, are among the 20% most efficient in their category.

### National consensus

All countries that wish to participate in the CDM should designate a national CDM body to evaluate and approve projects. This Designated National Authority is also the CDM focal point. Although in the framework of international processes, general guidelines are developed to determine the baseline conditions and the additional nature of the project activity, each developing country is responsible for developing national criteria for agreement on the project. Together with the investor, the host country should prepare the design and technical documentation with the following structure:

- General description of the project;
- Description of the methodology for determining the baseline;
- Schedule and crediting period;
- Methodology and monitoring plan;
- Calculation of GHG emissions by sources;
- Conclusion on environmental impact;
- Comments from stakeholders.

The designated national CDM authority issues the necessary conclusions that the government voluntarily participates in the project and confirms that the activities included in the project help the host country achieve sustainable development.

### Approval and registration

The designated operational entity (DOE) reviews the design documentation and, after receiving feedback and comments from the public, decides whether to approve it. These operating bodies will typically be private companies, such as audit and accounting firms, advisory firms or legal firms, which can independently and reliably estimate emission reductions. If the project is approved, the operating body sends the design and technical documentation to the Executive Board for official registration.

### Monitoring, verification, and certification

The carbon component of an emission reduction project cannot have real value in the international carbon market without going through a verification process that is specifically designed to quantify and verify the carbon component. Thus, when the project begins, its participants prepare a monitoring report that includes an assessment of the received certified emission reduction (CER) value and submit it to the operating body for verification. Verification is an independent ex-post assessment of the results of the emission reduction monitoring by the operating authority. The operating entity must ensure that the received CER volumes comply with the guidelines and conditions agreed upon during the initial approval of the project. After a detailed analysis, the operating entity issues a verification report and then certifies the CER volume obtained from the CDM project.

Certification is a piece of documentary evidence that the implementation of the project has resulted in proven emission reductions. Also, the certification report is an application for CERs. If a project participant or three members of the Executive Board do not request a review within 15 days, the Executive Board instructs the CDM Registry to issue CERs.

National benefit of CDM projects:

From the perspective of developing countries, the CDM can (UNCTAD 2003):

- Raise capital for projects that will help in the development of the economy while reducing carbon emissions;
- Allow and encourage the active participation of the public and private sectors;
- Become a tool for technology transfer if investments are directed to projects to replace obsolete and inefficient fossil fuel technologies or create new industries using environmentally sustainable technologies; and,
- Help to identify investment priorities for projects that meet the goals of sustainable development.

In particular, the CDM can contribute to the achievement of the sustainable development goals of the host countries by:

- Transfer of technology and financial resources;
- Sustainable energy production methods;
- Improving energy efficiency and energy savings;
- Poverty reduction through the creation of sources of income and new jobs;
- Positive environmental effects at the local level.

## Annex 2. CDM projects by countries

### The Republic of Albania

The Republic of Albania joined the United Nations Framework Convention on Climate Change (UNFCCC) in 1995. As a non-Annex I Party to the Convention, Albania achieved and offered its First National Communication at COP 8 in October 2002. The country completed the initial action in the process of the improvement of the Second National Communication by concluding the self-assessment activity and by creating the synthesis report on stocktaking of climate change activities. As a follow-up to the stocktaking exercise, Albania commenced the UNDP/GEF funded project for the preparation of the Second National Communication in March 2005. Albania ratified the KP in December 2004. The Designated National Authority was placed within the Climate Change Unit of the Ministry of Environment, Forests and Water Administration. Governmental rules and procedures for JI are not yet in place.

There is great potential for GHG remission, notably in the Albanian hydro sector. Apart from the 11 hydropower plants (HPPs) identified by Italy, there are more hydropower projects that could be used for CDM projects in the future, although the projects identified by Italy are the most flexible ones so far. According to Albania’s Second National Communication to the UNFCCC, total GHG emissions in 2005 reached 8.5 m t CO_2e_/year. Without extra measures to cut emissions, the GHG level was forecast to reach 37 m t CO_2e_ in 2020. Sectors with the highest emission reduction potential are the energy sector (400,000 t CO_2_/year), renewable energies (1.35 m t CO_2_/year), waste sector (130,000 t CO_2_/year), and LULUCF (620,000 t CO_2_/year). In sum, there are potential emission reductions of 2.5 m t CO_2_/year, 75% of which is accounted for by hydropower and forestry.

These days, there are various nations and foreign organizations involved in CDM projects and capability-building in Albania (Frasheri, 2013). Contracts were signed with Denmark and Italy. Key proposed deliverables of collaboration with Italy are the development of a set of rules and methods for CDM endorsement and DNA functioning, the advancement of standard baselines for energy and forestry sectors, and a national strategy for placing Albania in the carbon market. Italy has already supported the development of a DNA report that covers an analysis of existing institutional and juridical frameworks in the field of CDM. With Italian assistance, a local unit was founded in Tirana to aid the overall CDM activity in Albania (Austrian Development Agency, 2015). Italy also developed a portfolio of possible CDM projects (PIN status) in the field of energy efficiency and RES. With the Italian association, 11 projects have been developed in different sectors (3 waste management projects, 2 RES projects, 5 energy efficiency/fuel switch projects, and 1 afforestation project). The presentation of the CDM project portfolio took place in Milan, Italy, in May 2007. The Albanian Ministry for the Environment announced a call for tender for the feasibility investigations in September 2007.

The Austrian Development Assistance is also included in the CDM potential framework in Albania. It concentrates mostly on the same concerns as the Italian assistance. Austria has developed a report on CDM in the Albanian energy sector. There is greater focus on Austrian private companies, particularly for the development of the CDM projects in the field of construction and reconstruction of small hydropower plants. Moreover, as part of the UNDP/Austrian Government project “Capacity building to access carbon finance in Albania”, which was officially inaugurated in June 2007, a pipeline is currently being finished. No data about specific projects are currently available. The World Bank Carbon Fund has set up at least one project in Albania. The German development bank has also carried out an initial evaluation of a possible CDM project aimed at carbon sequestration through the natural restoration of forests around the Prespa Lake. Apart from the CDM activities, the UNDP and KfW are also active in the promotion of energy efficiency in Albania.

There is still a shortage of public controls and procedures for CDM project approval in Albania. The technological and commercial capability to develop PINs and PDDs is low. There is only limited information on the benefits of carbon finance to the economics of investment (IRR). Project owners need national data for the evaluation of baseline emissions. Efforts to ensure inclusion in carbon finance have emerged comparatively late compared to other countries, meaning that investors have shown insufficient interest in the country in the past.

### Bosnia and Herzegovina

Bosnia and Herzegovina has been a non-Annex I Party to the UNFCCC since December 2000 and ratified the KP in July 2007. The country has completely committed to satisfying the conditions of Art.4 and Art.12 of the Convention. The National Focal Point within the Ministry of Physical Planning, Civil Engineering and Ecology has made meaningful attempts to develop the Initial National Communication. It will also serve as the Designated National Authority. The CDM Board, however, is yet to be established.

Bosnia and Herzegovina’s energy sector is characterized by hydropower (Kaštelan-Macan et al. 2007) and coal, with total installed capacity of 1900 MW. The country is currently promoting an energy strategy that centres on HPPs, particularly on SHPPs. Bosnia and Herzegovina have implemented some legal authorizations regarding HPPs to encourage the growth of this type of project. Nevertheless, the known CDM projects are still at a very early stage. At the CTI-Investors Forum, Bosnia and Herzegovina presented two small-scale hydropower projects.

In 2003, Austria approved a Memorandum of Understanding (MoU) with Bosnia and Herzegovina and is involved in technical assistance for environmental projects. The World Bank is currently carrying supporting the development of the “Energy Study in BIH”, which will give recommendations for improving and sustaining the energy sector and assist Bosnia and Herzegovina with the founding of a national energy strategy.

So far, relatively few projects have been developed. However, interest in international cooperation and further project development was expressed at the CTI-Investors Forum. Capacity building among potential project owners is necessary to further develop Bosnia and Herzegovina’s project potential, and the founding of the DNA will be the crucial prerequisite for CDM activity.

### The Republic of Moldova

The Republic of Moldova ratified the UNFCCC in 1995 and the KP in 2003. The Moldovan DNA belongs to the State Hydrometeorological Service of the Ministry of Ecology and Natural Resources. CDM potential in Moldova has been identified, notably in the RES sector. Three CDM projects were enrolled in 2006, presented in association with the International Bank for Reconstruction and Development as the trustee of the Community Development Carbon Fund (CDCF) and the Netherlands.

The EU encouraged the improvement of CDM projects (Klepper and Peterson 2006) in the Republic of Moldova in the framework of a TACIS programme. Within that programme, some possible CDM projects were carried out in partnership with the State Hydrometeorological Service. One of these projects, a project on biogas from poultry farms, was presented at the CTI-Investors Forum. To date, the Republic of Moldova has approved an MoU with Denmark. CDM project potential in Moldova is insufficient but additional projects could be identified in the bioenergy field. The general investment climate seems to be advantageous, and investors at the CTI-Investors Forum appeared to be interested in the country’s projects.

### Serbia

The confirmation of a national cross-sectoral Climate Change Strategy and Action Plan will help Serbia to achieve a complete national strategic and legal framework for climate action (alleviation and accommodation) in agreement with international commitments and obligations on GHG mitigation (Paris Agreement and EU accession). The Republic of Serbia has been part of the UNFCCC since 2001 and the KP since 2008. The Ministry of Environmental Protection (MEP) is the national focal point for the implementation of the Convention and the Protocol.

To receive the Letter of Approval (LoA) from the Serbian government, the project owner should promote the project according to the special conditions set by the MEP, including the full package of documents along with the PDD. The project owner should present the extended project with the Determination Report to the MEP. If necessary, the MEP conducts an expert evaluation of the project to assess its compliance with MEP requirements. It evaluates the project and accompanying documents presented by the project owner within a month and, if the assessment is positive, issues an LoA. In the event of rejection, the ministry notifies the project owner in writing within a month, specifying the reasons for rejection. Regarding the general investment climate, since 2002, Serbia has ratified new legislation, regulations and procedures intended to improve the investment climate in its economy in general and the energy sector in particular. The Government’s attempts to improve the business environment and the investment climate in the country are beginning to bear fruit. Despite the political risks, investment has grown by almost 16% and is 4.5 times greater than 2002 levels, while foreign direct investment is currently at its highest level since 1991.

Serbia has enormous potential for reducing GHG (Komarov et al. 2012), in particular by improving energy efficiency and employing renewable energies. Serbia’s key energy policy responsibilities and preferences are established in the Energy Strategy for the Period until 2030, adopted by the Cabinet of Ministers in March 2006 (Dunjic et al. 2016). The Strategy starts out from the understanding that Serbia has limited conventional energy resources and thus has to rely on imports, and that it additionally suffers a lack of diversification of energy imports. For these reasons, the strategy highlights the value of conscious use of energy, an increase in domestic energy production, and a shift to alternative energy sources. Consequently, the Serbian government usually welcomes the development of CDM projects to realize this potential. The Energy Strategy anticipates the mass construction of wind power plants, which in turn are suitable for the CDM. Particular attention is paid to alternative energy sources, RES and biomass projects. The programme also allows for mass reconstruction of out-dated thermal power plants and combined heat and power plants. However, the strategy does not predict any financing and approval of programmes for specific projects. In short, therefore, it can be said that the Energy Strategy is currently only at the planning stage, without any real substance, and will not limit the opportunities to accomplish CDM projects in Serbia. For several potential CDM-project types, there are other programmes and laws adopted, which could influence the additionality issue of CDM projects. Nevertheless, in practice, the programmes have not provided financing for specific projects so far.

For the time being, Serbia’s CDM project portfolio consists of seven projects. The projects were identified in cooperation with foreign carbon funds and governments, e.g. United Kingdom of Great Britain and Northern Ireland, Liechtenstein and Italy. A Liechtenstein-Serbian CDM-Portfolio had been developed with four projects.

Serbia’s main requirements are the latest technology, highly professional labourers and technological support for the extension of standards and the creation of an internal market. Further foreign investment is needed to extend CDM projects and to identify potential projects. While Serbia has huge project potential, the framework for CDM projects in the country remains unproven, and the general investment climate is still difficult. Serbian authorities frequently seek to encourage foreign investment, and the wider public is well disposed to foreign investment. There are few restrictions on foreign ownership. However, both domestic and foreign investors still face challenges at a realistic level. These do not exactly relate to the issue of foreign ownership or investment, but rather to administrative hurdles that are arbitrarily enforced, or random delays.

### Montenegro

Montenegro became part of the UNFCCC on 27 January 2007 as a non-Annex 1 country. It ratified the KP in 2007. Since EU membership is a priority for the country, the alignment of Montenegrin law with the relevant parts of the *acquis communautaire* on the environment and climate change is an essential element of this transaction (Government of Montenegro 2017).

The modification to the Law on Environment in March 2007 and the Governmental decision of 1 June 2006 resulted in the establishment of the DNA. Montenegro’s DNA now resides within the Ministry of Sustainable Development and Tourism. The CDM procedures in Montenegro are in place. They specify that the letter of endorsement must be issued within 15 days after the submission of the PIN. The PDD must be developed or accepted no later than 30 days after submission (Ministry of Sustainable Development and Tourism of Montenegro, 2017).

Montenegro has one of the lowest levels of GHG emissions per unit of GDP in Central and Eastern Europe (Djurovic, 2017). The energy sector accounts for about 70% of total emissions. It is dominated by large hydropower and coal-based plants. The high carbon intensity makes Montenegro attractive for CDM. CDM projects are primarily feasible in the field of energy efficiency and RES. Montenegro currently has two projects considered for CDM: both of them are wind power plants. One project has been developed with Italy on a bilateral basis, and the other with the United Kingdom of Great Britain and Northern Ireland.

## Annex 3

Table 3

Table 4

**Table 3:** Entity list of the project “Moldova Biomass Heating in Rural Communities 2”

• Carbon Finance Unit Moldova	• Schweizerische
• EDP — Energias de Portugal, S.A., the Netherlands (production, transmission, distribution and supply of electricity) • Netherlands’ Ministry of Infrastructure and the Environment (IenM), the Netherlands • FUJIFILM Corporation, Japan (multinational photography and imaging company) • Idemitsu Kosan Co., Ltd., Japan (petroleum company) • JX Nippon Oil & Energy Corporation, Japan (energy) • The Okinawa Electric Power Co., Inc., Japan (energy) • Daiwa Securities Co. Ltd., Japan (Japanese investment bank) • Endesa Generacion, S.A., Spain (energy company) • Gas Natural SDG, S.A, Spain (energy, natural gas and electricity) • Kingdom of Spain — Ministry of Agriculture, Food and Environment and Ministry of Economy and Competitiveness, Spain • Hidroelectrica del Cantabrico, S.A., Spain (energy company) • Göteborg Energi AB, Sweden (energy company) • Government of Italy — Ministry for the Environment, Land and Sea, Italy • Government of Luxembourg — Ministry of the Environment, Luxembourg • Aalborg Portland A/S, Denmark (cement-producing company)	• Rückversicherungsgesellschafts AG (Swiss RE), Switzerland (a reinsurance company) • Maersk Olieog Gas AS, Denmark (oil and gas company) • Nordjysk Elhandel A/S, Denmark (an energy trading and management company) • DONG Naturgas A/S, Denmark (utilities, purchases and distributes natural gas) • Kommunalkredit Public Consulting GmbH, Austria (consulting company) • Brussels — Capital Region, Belgium • Kingdom of Belgium — Walloon Region Ministry of the Environment, Belgium • Statkraft Carbon Invest AS, Norway (utilities, generation, transmission, and/or distribution of electric energy) • Statoil ASA, Norway (energy company) • KfW Bankengruppe, Germany (German state-owned development bank) • BASF SE, Germany (German chemical company and the second largest chemical producer in the world)

**Table 4:** Entity List of the project “Moldova Energy conservation and GHG emissions reduction”

• Republic of Moldova (host country) — Carbon Finance Unit Moldova	• Japan — FUJIFILM Corporation (multinational photography and imaging company)
• Netherlands — International Bank for Reconstruction and Development (IBRD) as the Trustee of the Community Development Carbon Fund • Netherlands — Netherlands’ Ministry of Infrastructure and the Environment (IenM) • Denmark — Danish Ministry of Climate, Energy and Building/Danish Energy Agency • Denmark — DONG Naturgas A/S (utilities, purchases and distributes natural gas) • Denmark — Maersk Olie og Gas AS (oil and gas company) • Denmark — Nordjysk Elhandel A/S (is an energy trading and management company) • Austria — Kommunalkredit Public Consulting GmbH (consulting company) • Belgium — Brussels — Capital Region • Belgium — Kingdom of Belgium — Walloon Region Ministry of the Environment • Norway—Statkraft Carbon Invest AS (utilities, generation, transmission, and/or distribution of electric energy) • Norway — Statoil ASA (energy company) • Switzerland — Schweizerische Rückversicherungsgesellschafts AG (Swiss RE) (reinsurance and insurance) • Italy — Government of Italy, Ministry for the Environment, Land and Sea • Germany — KfW Germany BASF SE (German state bank)	• Japan — Idemitsu Kosan Co., Ltd. (oil company, oil refining company) • Japan — JX Nippon Oil & Energy Corporation (oil company) • Japan — The Okinawa Electric Power Co., Inc. (electric power company) • Japan — Daiwa Securities Capital Markets Co. Ltd. (financials, asset Management, investment Management) • Spain — Endesa Generacion, S.A. (energy company) • Spain — Gas Natural SDG, S.A. (energy, natural gas and electricity) • Spain — Hidroelectrica del Cantabrico, S.A • Spain Kingdom of Spain — Ministry of Agriculture, Food and Environment and Ministry of Economy and Competitiveness • Sweden — Göteborg Energi AB (energy company) • Luxembourg—Government of Luxembourg, Ministry of the Environment • Finland — Ruukki Metals Oy (basic resources, Metal industry)

## Annex 4 Methodology used (All methodologies approved and elaborated by the UNFCCC’s CDM)

I. **Moldova:**
  - Moldova Biomass Heating in Rural Communities 2 – *AMS-II.E. ver. 6 – Energy efficiency and fuel switching measures for building; AMS-III.B. ver. 6 – Switching fossil fuels*
  - Moldova Energy conservation and greenhouse gases emissions reduction—*AMS-II.E. ver. 6 – Energy efficiency and fuel switching measures for building; AMS-III.B. ver. 6 – Switching fossil fuels*
  - Reducing gas leakages in Moldovagaz distribution network, Republic of Moldova – *AM0023 ver.4—Leak detection and repair in gas production, processing, transmission, storage and distribution systems and in refinery facilities*
  - Moldova Community Forestry Development Project – *AR-AM0002 ver.3—Restoration of degraded lands through afforestation/reforestation*
II. **Montenegro:**
  - Hydropower Plant Otilovici—*AMS-I.D. ver. 17 – Grid-connected renewable electricity generation*
  - b) Mozura Wind Farm—*ACM0002 ver. 12—Consolidated baseline methodology for grid-connected electricity generation from renewable sources*
III. **Bosnia and Herzegovina:**
  - Amitea Small Hydro Project—*AMS-I.D. ver. 17 – Grid-connected renewable electricity generation*
  - Hydro Power Plant Ulog—*ACM0002 ver. 13—Consolidated baseline methodology for grid-connected electricity generation from renewable sources*
IV. **Albania:**
  - HPP Ashta—*ACM0002 ver. 12—Consolidated baseline methodology for grid-connected electricity generation from renewable sources*
  - b) Devoll Hydropower (DHP)—*ACM0002 ver. 12—Consolidated baseline methodology for grid-connected electricity generation from renewable sources*
  - c) Hydropower station Murdhari 1&2 (Hydroelectric Power Station Murdhari in Albania)—*AMS-I.D. ver. 17 – Grid-connected renewable electricity generation*
V. **Serbia:**
  - Wind Farm Cibuk 1—*ACM0002 ver. 12—Consolidated baseline methodology for grid-connected electricity generation from renewable sources*
  - Wind Farm Plandiste 1—*ACM0002 ver. 12—Consolidated baseline methodology for grid-connected electricity generation from renewable sources*
  - Wind Farm Kosava I + II—*ACM0002 ver. 12—Consolidated baseline methodology for grid-connected electricity generation from renewable sources*
  - Wind Farm Kladovo 1- *ACM0002 ver. 12—Consolidated baseline methodology for grid-connected electricity generation from renewable sources*
  - LFG Recovery and Electricity Production at the Bubanj Landfill Site, Nis, Serbia—*AMS-III.G. ver. 7—Landfill methane recovery; AMS-I.D. ver. 17 – Grid-connected renewable electricity generation*
  - Alibunar Biogas plant construction project—*AMS-III.AO.—Methane recovery through controlled anaerobic digestion; AMS-I.D. ver. 17 – Grid-connected renewable electricity generation*

Table 5

**Table 5:** Categories of methodologies and their characteristics

Approved full-blown methodologies (AM)	Approved consolidated methodologies (ACM)	Approved small-scale methodologies (SSC)
• The largest group of methodologies; • Initially developed by project participants for a specific project, but then can be used for other similar projects that meet certain conditions of applicability; • Usually does not have an upper limit on the size or capacity of plants and the reduction of emissions; • More comprehensive than small-scale ones; • A stricter emphasis has been placed on monitoring compared to small-scale ones	• Combines a number of full-blown methodologies for similar or related project types into one methodology; • Association by the Expert Group on Methodologies under the UNFCCC and not by project participants; • Wider focus / less for a single project	• Applicable small-scale projects cannot exceed a certain threshold (for example, determined on the basis of capacity for generating electricity, conserving energy, or reducing emissions) • Compared to full-blown methodologies, SSC methodologies have the following advantages: 1) Identical project components can be grouped as part of a single project activity; 2) Baseline calculations; 3) Monitoring procedures are simplified to reduce costs; 4) The same DOE can carry out both validation and verification of the project

## Annex 5

Table 6

**Table 6:** Parties included in the financing of the CDM project

Organization	Role/responsibility
Project host	The host of a project is an organization that provides the land, capacity, or resources required to complete the CDM project in a developing country — the location of the project. In principle, there can be more than one project host, for example, for a wind farm project, one side can be the landowner and the other can install and own wind turbines. Individuals, companies, and government organizations may host the project
CDM project developer	The CDM project developer is the organization responsible for passing the project through the CDM project cycle. The project host may assume this role, or a development company specializing in CDM projects may provide it
CDM project participant	“CDM project participant” has special meaning in the framework of the CDM. A project participant is either a Party to the KP (for example, a government) involved in the project, or a private company authorized by the involved Party to participate in the project. Project participants can only make the decision on the distribution of CERs received in the project. Project participants may agree among themselves (and declare in a document filled out by the CDM Executive Board during registration, known as the Terms of Communication) that one or more project participants are the coordinator(s). In this case, only the coordinator(s) decide(s) how to distribute CERs from the project
Project coordinator	A CDM project coordinator is a project participant or participants named in the Communication Conditions as a project coordinator
Buyers of CERs issuance	Theoretically, any organization can buy the CERs received in the project. However, to enable the use of CERs to comply with obligations under the KP or any system with obligations related to the KP, the buyer of CERs should be either the Party of Annex 1 or the company of Annex 1 authorized by the Authorized National Authority to be able to transfer CERs from the CDM project to the account registered in the country of the buyer
Authorized operational authority	Required for validation of the project prior to its registration as a CDM project, and verification of the reduction of emissions in the project before the issuance of CERs. In essence, the AOA plays the role of an independent auditor
Designated national authority	In the developing country in which the project is located, the project must be approved (by issuing a Letter of Approval) before validation. The DNA are required for the approval of any project participants from Annex 1 countries
CDM Executive Board	The CDM Executive Board is responsible for administering procedures related to project registration and issuance of CERs

## Annex 6

Costs play a major role in the study of our projects, since in addition to the costs that the project incurs regardless of whether it will be registered as a CDM or not, some specific costs are associated with different stages of the CDM project cycle, as shown in Table 7 below.

**Table 7:** CDM-specific project costs

Activities	Cost (full-blown, US $)	Cost (small scale, US $)	Cost type
Planning phase			
Initial feasibility study i.e. Design Concept (PIN)	5000–30,000	2000–7500	Consulting services or internal
Project Design Document	15,000–100,000	10,000–25,000	Consulting services or internal
New methodology (if required)	20,000–100,000 (including 1000 US dollars—registration UN contribution fees)	20,000–50,000	Consulting services or internal
Validation	8000–30,000	6500–10,000	Payment to an Authorized Operational body
Registration fee	10 500–350,000^5^	0–24,500^6^	Contribution to the CDM Executive Board
Total specific costs for the CDM—planning phase	**38,500**–**610,000**	**18,500**–**117,000**	
Construction phase			
Construction, manufacturing and equipment	Variable, depending on the type of project		Builders payment
Installation of monitoring equipment	Usually minimal in relation to the total Production costs and equipment		Builders payment
Total specific costs for the CDM—construction phase	**Usually minimal in relation to the total Production costs and equipment**		
Execution phase			
Contribution to the UNDP Adaptation Fund	2% CER	2% CER	Executive Board Contribution
Initial verification (incl. System check)	5000–30,000	5000–15,000	Payment to an Authorized Operational Authority
Current verification (periodic)	5000–25,000	5000–10,000	Payment to an Authorized Operational body
Share of administrative expenses (SOP-Admin.)	The registration fee is actually paid in advance, and will be checked against the CERs actually put into circulation during the crediting period (if there is a difference with respect to the forecast reduction during registration). SOP admin. not covered		Executive Board Contribution
Total CDM-specific cost—implementation phase	**Changes—minimum 2% of CERs plus 5000 / year (if verification is performed annually)**		

^1^US $ 0.10 / tonne of CERs for the first 15,000 CERs per year; and US $ 0.20 / tonne of CERs for CERs over 15,000 per year (maximum US $ 350,000). The minimum shown here is calculated as 15,000 CERs / year for one lending period of 7 years.

2As for full-scale projects, if the total annual reduction in emissions is below 15,000 tonnes of CO_2_ eq., the registration fee is not paid. Maximum estimated 25,000 CERs / year for 7 years of the loan period.

## Annex 7

Table 8

Table 9

**Table 8:** General types of financing CDM projects

Grants	Loans (debt)	Equity
A grant is the amount of currency presented by a third party to a project, individual, or organization to contribute to the objectives of the third party. As a rule, grants are provided to commercially low income projects, and there is no need for the grant to be returned (provided that the stated purpose of financing the grant has been achieved). However, in some cases, if the project achieves commercial success, grants may turn into loans or ownership, (if so, this must be stated in the terms and conditions of the grant). As a rule, grants are issued by state structures and cover only a percentage of the costs of the project, meaning other forms of financing are also needed	A loan or debt is the amount of money provided by a third party to a project, individual, or organization that must be repaid during or at the end of the agreed period, plus a percentage during the loan period. Banks provide most loans for projects *1) Principal loans or debts;* *2) Subordinate (or secondary) loans or debts;* *3) Loans or debt at a low-interest rate;* *4) Advance payments;* *5) Leasing financing*	Ownership is equity received from shareholders. The shareholders have only the residual amount of the claim concerning the assets of the project company; in other words, they are in the queue for the repayment of debts after other interested parties, such as main and lower creditors. This represents the highest level of risk, and the expected return for holders of equity is correspondingly higher than for lenders. For the project developer, equity offers the advantage of not having to return the money, thus freeing up cash, which is often important, especially during the first years of the project

**Table 9:** Risks during various phases of the implementation of CDM projects

Planning phase	Construction phase	Execution phase
Feasibility risk	The risks of time overrun	Technological risk
Permission/licence risk	Capital overrun risk cost	Market risk
		Supply risk
		Operational risk
		Political, legal and regulatory risks
		Financial risk
		Affiliate risk

## Footnotes

1 The CDM, which is defined in Article 12 of the KP, seeks to help developing countries to achieve sustainable development by supporting environmentally-friendly investments from the government and the private sector of industrialized countries. The CDM financing should help developing countries meet a range of economic, social, environmental and sustainable development goals, such as ensuring clean air and water, improving land use, rural development, increased employment, reduction of poverty and, in many cases, reduction of dependence on imported fossil fuels (UN 2011; UNFCCC 2009).

2 SDG7 calls for efforts to ensure access to affordable, reliable, sustainable and modern energy for all. SDG13 calls for urgent action to combat climate change and its impacts.

3 An exhaustive list of CDM projects analysed in Li and Lin (2021) did not pay specific attention to those located in Eastern Europe.

4 A recent report on SDG performance focused on Italy is provided by D’Adamo et al. (2021).

## References

[CR1] Acerbi F, Sassanelli C, Terzi S, Taisch M (2021). A systematic literature review on data and information required for circular manufacturing strategies adoption. Sustainability.

[CR2] Alberola E, Chevallier J, Cheze B (2008). Price drivers and structural break in European carbon prices 2005–07. Energy Policy.

[CR3] Anger N, Bohringer C, Moslener U (2007) Macroeconomic impacts of the CDM: The role of investment barriers and regulations, ZEW discussion paper No. 07–026 Mannheim

[CR4] Begić F, Afgan N (2007). Sustainability assessment tool for the decision making in selection of energy system—Bosnian. Energy.

[CR5] Benites-Lazaro LL, Gremaud PA, Benites LA (2018). Business responsibility regarding climate change in Latin America: an empirical analysis from Clean Development Mechanism (CDM) project developers. The Extractive Industries and Society.

[CR6] Bossink (2017). Demonstrating sustainable energy: a review based model of sustainable energy demonstration projects. Renew Sustain Energy Rev.

[CR7] Burian M (2006) The clean development mechanism, sustainable development and its assessment, Hamburg Institute of International Economics

[CR8] Burniaux JM, Chateau J, Dellink R, Duval R, Jamet S (2009) The economics of climate change mitigation: how to build the necessary global action in a cost-effective manner, Economics department working papers No.701 OCDE

[CR9] Burtraw AM, Kruger D, Zetterberg L (2007). The ten-year rule-allocation of emissions allowances in the EU emissions trading system. Energy Policy.

[CR10] Cassimon D, Prowse M, Essers D (2014). Financing the clean development mechanism through debt-for-efficiency swaps?. Case Study Evidence from a Uruguayan Wind Farm Project, the European Journal of Development Research.

[CR11] Chao Q, Feng A (2018). Scientific basis of climate change and its response. Global Energy Interconnection.

[CR12] Convery FJ (2009). Reflections-the emerging literature on emissions trading in Europe. Rev Environ Econ Policy.

[CR13] Criqui P, Kitous A (2003) Impacts of linking JI and CDM credits to the EU ETS. Kyoto Protocol Implementation, KPI, Technical Report, B4–3040/2001/330760/MAR/E1

[CR14] Cui J, Liu X, Sun Y, Yu H (2020). Can CDM projects trigger host countries’ innovation in renewable energy? Evidence of firm-level dataset from China. Energy Policy.

[CR15] Cvetković S, Kaluderovic T, Vulkadinovic B, Kijevanin M (2014). Potentials and status of biogas as energy source in the Republic of Serbia. Renew Sustain Energy Rev.

[CR16] Das K (2011) Technology transfer under the clean development mechanism: an empirical study of 1000 CDM projects. Available at SSRN: https://ssrn.com/abstract=1887727 or 10.2139/ssrn.1887727

[CR17] Dixon T, Romanak K, Neades S, Chadwick A (2013). Getting science and technology into international climate policy: carbon dioxide capture and storage in the UNFCCC. Energy Procedia.

[CR18] Dixona T, Leamonb G, Zakkourc P, Warrend L (2013). CCS Projects as Kyoto Protocol CDM activities. Energy Procedia.

[CR19] Djurovic G, Bigovic M, Milovic N (2016). Support for further enlargement of the EU: statistical analysis of regional differences. J Balkan near East Stud.

[CR20] Djurovic G (2017) Building a sustainable future for montenegro through the EU accession process and the UN sustainable development goals. Montenegro: UNDP, pages 6–10

[CR21] Djurovic G, Muhadinovic M, Djurovic V, Bojaj M (2019). Agenda 2030: measuring progress in the Montenegro’s National Strategy for Sustainable Development through SDG indicators.

[CR22] Dogmus OØ, Nielsen J (2019). Is the hydropower boom actually taking place? A case study of a South East European country, Bosnia and Herzegovina. Renew Sustain Energy Rev.

[CR23] Dong F, Gao Y, Li Y, Zhu J, Hu M, Zhang X (2022). Exploring volatility of carbon price in European Union due to COVID-19 pandemic. Environ Sci Pollut Res.

[CR24] Dunjic S, Pezzutto S, Zubaryeva A (2016). Renewable energy development trends in the Western Balkans. Renew Sustain Energy Rev.

[CR25] D’Adamo I, Gastaldi M, Imbriani C, Morone P (2021). Assessing regional performance for the Sustainable Development Goals in Italy. Sci Rep.

[CR26] Frasheri A (2013) Geothermal energy resources in Albania-Country update paper, European Geothermal Congress 2013 Pisa, Italy

[CR27] Gaast W, Begg K (2009). Promoting sustainable energy technology transfers to developing countries through the CDM. Appl Energy.

[CR28] Government of the Republic of Moldova (2017) Adapting the 2030 agenda on sustainable development to the context of the Republic of Moldova. https://statistica.gov.md/public/files/SDG/docs/Targets_ONU_EN.pdf

[CR29] Government of Montenegro (2017) Montenegro’s development directions 2018–2021 [Internet]. Available from: https://www.gov.me/

[CR30] Zhang C, Wu Y, Yang Y (2018). The influencing factors of sCER price dynamics under the clean development mechanism: theory and econometric analysis. J Syst Sci Complexity.

[CR31] Zhou Y, Chen X, Tan X, Liu C, Zhang S, Yang F, Zhou W, Huang H (2018). Mechanism of CO2 emission reduction by global energy interconnection. Global Energy Interconnection.

[CR32] Gribincea C (2013) Energy efficiency policy in Moldova, Scientific Papers Series Management, Economic Engineering in Agriculture and Rural Development 13(1)

[CR33] Hawkes AD (2014). Long-run marginal CO2 emissions factors in national electricity systems. Appl Energy.

[CR34] Hepburn CJ (2007). Carbon trading: a review of the Kyoto mechanisms. Annu Rev Environ Resour.

[CR35] Huang Y, Barker T (2012). The clean development mechanism and low carbon development: a panel data analysis. Energy Economics.

[CR36] Huang W, Wang Q, Li H, Fan H, Qian Y, Klemeš JJ (2022) Review of recent progress of emission trading policy in China. J Clean Prod, 131480

[CR37] Jahanger A, Usman M, Murshed M, Mahmood H, Balsalobre-Lorente D (2022). The linkages between natural resources, human capital, globalization, economic growth, financial development, and ecological footprint: the moderating role of technological innovations. Resour Policy.

[CR38] Jiang T, Yu Y, Jahanger A, Balsalobre-Lorente D (2022) Structural emissions reduction of China’s power and heating industry under the goal of “Double Carbon”: a perspective from input-output analysis. Sustainable Production and Consumption

[CR39] Jotzo F, Michaelowa A (2002). Estimating the CDM market under the Marrakech accords. Climate Policy.

[CR40] Karaj Sh, Rehl T, Leis H, Muller J (2010). Analysis of biomass residues potential for electrical energy generation in Albania. Renew Sustain Energy Rev.

[CR41] Karakosta C, Flouri M, Dimopoulou S, Psarras J (2012). Analysis of renewable energy progress in the western Balkan countries: Bosnia-Herzegovina and Serbia. Renew Sustain Energy Rev.

[CR42] Kaštelan-Macan M, Ahel M, Horvat A, Jabucar D (2007). Water resources and waste water management in Bosnia and Herzegovina. Croatia and the State Union of Serbia and Montenegro, Water Policy.

[CR43] Kiel Institute for World Economics (2004) Lückge H., Peterson S. The role of CDM and JI for fulfilling the European Kyoto commitments, Kiel Working Paper, 1232

[CR44] Kirkman G, Seres S, Haites E (2013). Renewable energy: comparison of CDM and Annex I projects. Energy Policy.

[CR45] Klepper G, Peterson S (2006). Emissions trading, CDM, JI, and more: the climate strategy of the EU. Energy J.

[CR46] Komarov D, Stupar S, Simonovic A, Stanojevic M (2012). Prospects of wind energy sector development in Serbia with relevant regulatory framework overview. Renew Sustain Energy Rev.

[CR47] Koondhar MA, Shahbaz M, Memon KA, Ozturk I, Kong R (2021). A visualization review analysis of the last two decades for environmental Kuznets curve “EKC” based on co-citation analysis theory and pathfinder network scaling algorithms. Environ Sci Pollut Res.

[CR48] Kostakis I, Tsagarakis KP (2022) The role of entrepreneurship, innovation and socioeconomic development on circularity rate: empirical evidence from selected European countries. J Clean Prod, 131267

[CR49] Lalic D, Popovski K, Gecevska V, Popovska-Vasilevska S, Tesic Z (2011). Analysis of the opportunities and challenges for renewable energy market in the Western Balkan countries. Renew Sustain Energy Rev.

[CR50] Lee J, Jang H (2022) A real options study on cook stove CDM project under emission allowance price uncertainty. J Asian Econ, 101464

[CR51] Lema A, Lema R (2013). Technology transfer in the clean development mechanism: insights from wind power. Glob Environ Chang.

[CR52] Li Z, Lin B (2021). What matters in the distributions of clean development mechanism projects? A panel data approach. Environ Impact Assess Rev.

[CR53] Liu L, Stephane K, Zhang W (2018). Country variation of clean development mechanism project registration time: an exploratory analysis by the Cox model. Procedia Computer Science.

[CR54] Liu L, Zhang M, Zhao Z (2019). The application of real option to renewable energy investment: a review. Energy Procedia.

[CR55] Lowitzsch J, Hoicka E, van Tulder J (2020). Renewable energy communities under the 2019 European Clean Energy Package – governance model for the energy clusters of the future?. Renew Sustain Energy Rev.

[CR56] Manton MJ, Belward A, Harrison D, Kuhn A, Lefale P, Rosner A, Simmons A, Westermeyer W, Zilman J (2010). Observation needs for climate services and research. Procedia Environ Sci.

[CR57] Mansanet-Bataller M, Chevallier J, Hervé-Mignucci M, Alberola E (2011). EUA and sCER phase II price drivers: unveiling the reasons for the existence of the EUA–sCER spread. Energy Policy.

[CR58] Mendez-Sayago JA, Perugache-Rodriguez CA (2012). Causality and sensitivity of prices of European Union allowances and emission reduction certificates of clean development mechanisms in the European market for allowance transactions. Estudios Gerenciales.

[CR59] Michaelowa A, Dutschke M (2002) Integration of climate and development policies through the clean development mechanism, EADI, GEMDEV (eds.): Europe and the South in the 21st century, Challenges for renewed cooperation, Karthala, Paris

[CR60] Mikicic D, Radicevic B, Ðurisic Z (2006). Wind energy potential in the world and in Serbia and Montenegro, FACTA UNIVERSITATIS (NIS) SER.: ELEC. Energ.

[CR61] Ministry of Sustainable Development and Tourism of Montenegro (2017) The information about initial results in the NSSD 2030 in the period July 2016–December 2017, Document prepared for the National Council for SDCCICZM

[CR62] Nikolakakis T, Chattopadhyay D, Malovic D, Vayrynen J, Bazilian M (2019). Analysis of electricity investment strategy for Bosnia and Herzegovina. Energ Strat Rev.

[CR63] Olsen K (2007). The clean development mechanism’s contribution to sustainable development: a review of the literature. Clim Change.

[CR64] Austrian Development Agency (2015) Albania: country strategy 2015–2020 *extended until the end of 2012. Federal Ministry for Europe, Integration and Foreign Affairs Directorate General for Development Cooperation.https://www.entwicklung.at/fileadmin/user_upload/Dokumente/Publikationen/Landesstrategien/CS_Albania_2015-2021.pdf

[CR65] Rickerson W, Perroy R (2005) Renewable energy development on the edge of the European Union: a case study of Albania, Association of American Geographers 2005 Conference, Denver, CO

[CR66] Ruthner L, Johnson M, Chattejee B, Lazarus M, Fujiwara N, Egenhofer C, Monceau T, Brohe A (2011) Study on the integrity of the clean development mechanism (CDM), European Commission report

[CR67] Shankar A, Quinn A, Dickinson K, Williams K, Masera O, Charron D, Jack D, Hyman J, Pillarisetti A, Bailis R, Kumar P, Ruiz-Mercado I, Rosenthal J (2020). Everybody stacks: lessons from household energy case studies to inform design principles for clean energy transitions. Energy Policy.

[CR68] Tang W, Du S, Hu L, Wang B, Zhu Y (2022). The effects of leadership in clean development mechanism low-carbon operations. Transport Res E Log Transport Rev.

[CR69] Tešić M, Kiss F, Zavargo Z (2011). Renewable energy policy in the Republic of Serbia. Renew Sustain Energy Rev.

[CR70] The Danish Institute for Human Rights (n.d) Available at https://sdg.humanrights.dk/en/goals-and-targets (Accessed, Apr, 26^th^ 2022).

[CR71] ŢÎŢEI V (2002) The evaluation of biomass of the Sida hermaphrodita and Silphium perfoliatum for renewable energy in Moldova, Botanical Garden (Institute) of the Academy of Sciences of Moldova

[CR72] Udovicki K, Sormaz N, Babic D, Urosev A, Colic V, Pejcic M, lazarevic J, Medic P (2018) Serbia sustainable development issues: a baseline review, Center of Advanced Economic Studies (CEVES), Belgrade

[CR73] Ugochukwu B (2020). Challenges of integrating SDGs in market-based climate mitigation projects under the Paris agreement. McGill Int J Sustain Dev Law Policy.

[CR74] UN (2011) Annual report of the executive board of the clean development mechanism to the conference of the parties serving as the meeting of the parties to the Kyoto Protocol, clean development mechanism, Executive board, Geneva: UN, 2011

[CR75] UNCTAD (2003) An implementation guide to the clean development mechanism. (https://unctad.org/en/Docs/ditcted20031_en.pdf)

[CR76] UNEP (2004) CDM information and guidebook (second edition), United Nations Environment Programme, Roskilde, Denmark

[CR77] UNFCCC (2009) CDM statistics, United Nations Framework Convention on Climate Change. Available at: http://ghg.unfccc.int

[CR78] UNFCCC (2012) Benefits of the clean development mechanism. (https://cdm.unfccc.int/about/dev_ben/ABC_2012.pdf)

[CR79] Usman M, Balsalobre-Lorente D (2022). Environmental concern in the era of industrialization: can financial development, renewable energy and natural resources alleviate some load?. Energy Policy.

[CR80] Vasa A (2012). Certified emissions reductions and CDM limits: revenue and distributional aspects. Climate Policy.

[CR81] Xhitoni A (2013). Renewable energy scenarios for Albania.

[CR82] Zakkour P, Scowcroft J, Heidug W (2014). The role of UNFCCC mechanisms in demonstration and deployment of CCS technologies. Energy Procedia.

[CR83] Zhang C, Yan J (2015). CDM’s influence on technology transfers: a study of the implemented clean development mechanism projects in China. Appl Energy.

